# Exacerbations in COPD Patients with Bronchiectasis

**DOI:** 10.3390/medsci5020007

**Published:** 2017-04-11

**Authors:** Jordan Minov, Saso Stoleski, Dragan Mijakoski, Kristin Vasilevska, Aneta Atanasovska

**Affiliations:** 1 Institute for Occupational Health of R. Macedonia—WHO Collaborating Center, 1000 Skopje, Macedonia; sstoleski@yahoo.com (S.S.); dmijakoski@yahoo.com (D.M.); aneta.atanasovska@yahoo.com (A.A.); 2 Institute for Epidemiology and Biostatistics, 1000 Skopje, Macedonia; vasile_kris@yahoo.com

**Keywords:** Bronchiectasis, COPD, exacerbation, HRCT

## Abstract

There is evidence that coexisting bronchiectasis (BE) in patients with chronic obstructive pulmonary disease (COPD) aggravates the course of the disease. In this study, we aimed to evaluate the frequency and severity of bacterial exacerbations in COPD patients with BE. The frequency and duration of bacterial exacerbations treated in a 12-month period, as well as the duration of the exacerbation-free interval, were evaluated in 54 patients with COPD (Group D) who were diagnosed and assessed according to official recommendations. In 27 patients, BE was diagnosed by high-resolution computed tomography (HRCT), whereas an equal number of COPD patients who were confirmed negative for BE by HRCT, served as controls. We found a significantly higher mean number of exacerbations in a 12-month period in COPD patients with BE (2.9 ± 0.5), as compared to their mean number in controls (2.5 ± 0.3) (*p* = 0.0008). The mean duration of exacerbation, i.e., the mean number of days elapsed before complete resolution of the symptoms or their return to the baseline severity, was significantly longer in COPD patients with BE as compared to their mean duration in controls (6.9 ± 1.8 vs. 5.7 ± 1.4; *p* = 0.0085). In addition, the mean exacerbation-free interval expressed in days, in patients with COPD with BE, was significantly shorter than in COPD patients in whom BE were excluded (56.4 ± 17.1 vs. 67.2 ± 14.3; *p* = 0.0149). Overall, our findings indicate that coexisting BE in COPD patients may lead to more frequent exacerbations with a longer duration.

## 1. Introduction

Bronchiectasis (BE) is characterized by irreversible widening of the medium-sized airways, along with inflammation, chronic bacterial infection, and destruction of the bronchial walls. BE represents a final common pathway for a large number of disorders, such as development of BE following severe pneumonia, infection with *Mycobacterium tuberculosis*, or with non-tuberculous mycobacteria (NTM), and allergic bronchopulmonary aspergillosis (ABPA). BE may be associated with connective tissue diseases (e.g., rheumatoid arthritis), inflammatory bowel disease (IBD), or with inhaled foreign bodies, lung tumors, and other obstructive lesions. In addition, there are a number of congenital or inherited causes of BE, most of which are rare. A large percentage of all BE cases (20%–60%) have unknown causes, and are considered to be idiopathic [1].

With the widespread availability of high-resolution computed tomography (HRCT), many patients with BE have been detected. It has been estimated that at the beginning of this century, there have been at least 110,000 adults in the USA with BE [2]. However, the current incidence and prevalence of BE is still not clear, and existing evidence indicates a huge variability in BE prevalence, depending on the geographic area considered. In countries as Korea, which has a high tuberculosis incidence rate, the prevalence of BE in the general population is high, i.e., in a study on 1409 Korean adults, Kwak et al. [3] diagnosed BE in 9.1% of the study subjects based on findings from chest computed tomography (CT) scans. In addition, Weycker et al. [2] estimated the prevalence of BE to be 52.3 cases per 100,000 adults in the US from a study with a retrospective cohort design which included more than 56 million patients from multiple US health plans. More recently, Quint et al. [4,5] reported an increase in point prevalence of BE in the UK in women, from 350.5 cases per 100,000 in 2004 to 566.1 per 100,000 in 2013, and in men from 301.2 cases per 100,000 in 2004 to 485.5 per 100,000 in 2013.

Similar to the clear overlap between chronic obstructive pulmonary disease (COPD) and asthma, termed the asthma-COPD overlap syndrome (ACOS), there is also a clear overlap between BE and COPD [5,6]. According to some findings, up to 50% of patients with moderate to severe COPD had coexisting BE [7,8]. Furthermore, in a population-based study performed in the UK, Quint et al. found that 42.5% of patients with BE had a coexisting diagnosis of asthma, and 36.1% had a coexisting diagnosis of COPD [4,5].

COPD and BE share many pathophysiological and clinical characteristics. In addition, findings of BE from HRCT scans in patients with COPD indicates the presence of more advanced airway dysfunction, bacterial colonization and frequent exacerbation [9,10]. In the 2014 Global Initiative for Chronic Obstructive Lung Disease (GOLD) guidelines, BE was for the first time defined as a comorbidity of COPD, and this change was retained in the 2015 update [11,12].

The aim of the present study was to evaluate the frequency and duration of bacterial exacerbations, as well as the duration of the exacerbation-free interval in COPD patients with coexisting BE.

## 2. Materials and Methods

### 2.1. Study Design and Setting

The study was designed as a comparison of frequency and duration of bacterial exacerbations, as well as comparing the duration of exacerbation-free intervals between a group of COPD patients with BE and a group of COPD patients who were confirmed negative for BE. The study was performed from December 2015 to December 2016 by the Institute for Occupational Health, R. Macedonia, Skopje (a referral institution with five pulmologists working with outpatients, approximately 6000 respiratory patients per year).

### 2.2. Study Subjects

The study population included 27 patients with COPD group D (15 males and 12 females, aged 43 to 61 years) with BE diagnosed by HRCT, and an equal number of Group D COPD patients with similar demographic characteristics (16 males and 11 females, aged 44 to 62 years), who were confirmed negative for BE through HRCT. We opted to investigate COPD patients from Group D as the existing evidence indicates that a greater severity of functional impairment in COPD patients is accompanied by a higher prevalence of BE [5,7,10].

Patients with a history of asthma, lung cancer, or other significant respiratory disease, as well as those unable to complete diary cards, were excluded from the study. All study subjects were recruited in the stable phase of the disease, i.e., without any evidence of exacerbation for at least three weeks.

Daily stable respiratory symptoms (baseline symptoms), medication use, and history of exacerbations, were noted in all subjects before they commenced the study. All study subjects underwent baseline and post-bronchodilator spirometry, according to the official recommendations of the European Respiratory Society (ERS) and the American Thoracic Society (ATS) [12,13]. In addition, in all study subjects a microbiological evaluation of sputum was performed, according to official recommendations [14].

The Body Mass Index (BMI), a measure of body fat based on adult height and weight, was determined in all study subjects by computed calculation using a BMI calculator [15].

Classification of smoking status was conducted according to World Health Organization (WHO) recommendations [16]. Passive smoking or exposure to environmental tobacco smoke (ETS) was defined as an exposure to tobacco combustion products from smoking by others (at home, workplace, etc.), i.e., a presence of at least one smoker in the household and/or in the workplace [17,18].

### 2.3. Ethics Statement

All study subjects were informed about the study and their written consent was obtained. The Ethical Committee of the Institute of Occupational Health of R. Macedonia, Skopje—WHO Collaborating Center and GA2LEN Collaborating Center gave signed approval for performing the study and publishing the results obtained (03-714/31.08.2015).

### 2.4. Diagnosis and Assessment of Chronic Obstructive Pulmonary Disease

According to the official GOLD recommendations, COPD was considered by identifying a post-bronchodilator ratio between forced expiratory volume in one second, and a forced vital capacity (FEV_1_/FVC ratio) less than 0.70, in symptomatic subjects (with dyspnea, chronic cough, or sputum production) with a history of exposure to risk factors for the diseases (noxious particles and gases).

Subjects with diagnosed COPD were classified according to the combined COPD assessment which included the assessment of symptoms, degree of airflow limitation, and risk of exacerbations. COPD patients classified as a Group D were characterized by frequent symptoms (overall score of the COPD Assessment Test (CAT) equal or higher than 10), severe or very severe airflow limitation (FEV_1_ value ranging from 30% to 50% of its predicted value, or less than 30% of its predicted value) and high risk of exacerbation (two or more exacerbations per year, or one or more exacerbations requiring hospitalization per year).

During the study period, all study subjects were given regular treatment for stable disease status in accordance with official GOLD recommendations [11,12].

### 2.5. Diagnosis of Bronchiectasis

Diagnosis of BE was based on the findings of HRCT as it is currently considered to be the best tool for diagnosis of BE. All study subjects underwent the same HRCT scan procedure. In addition, all scans were interpreted independently by both a radiologist and a member of the study team.

According to the actual recommendations, the main diagnostic features for BE were a bronchus internal diameter that was wider than its adjacent blood vessel, a failure of the bronchi to taper, and visualization of the bronchi in the outer 1–2 cm of the lung fields [7,19,20,21]. BE was scored in each lobe by consensus, using the grading system proposed by Smith et al. [22] as follows: 0 if no BE was present; 1 if less than 25% of the bronchi were bronchiectatic; 2 if 25%–49% of the bronchi were bronchiectatic; 3 if 50%–74% of the bronchi were bronchiectatic; and 4 if 75% or more of the bronchi were bronchiectatic. As previous studies showed that more than 50% of healthy volunteers may have at least one dilated bronchus on HRCT [23], only patients with a total BE score of 2 or more were considered to have changes consistent with clinically significant disease, for the purposes of the study objectives.

### 2.6. Diagnosis and Treatment of Chronic Obstructive Pulmonary Disease Exacerbation

According to the actual GOLD recommendations, COPD exacerbations were considered as acute events characterized by a worsening of the patient’s respiratory symptoms that was beyond normal day-to-day variations, and led to a change in medication. As the most common causes of exacerbations are viral and bacterial respiratory infections, and because there is currently no biomarker allowing precise etiologic diagnosis, the diagnosis of exacerbation was defined by the patient’s symptoms, using the criteria described by Anthonisen et al. [24]. Probable bacterial aetiology was established when the exacerbation was Anthonisen Type I (presence of three cardinal symptoms: increased dyspnea, sputum volume, and purulence), or Type II (presence of two cardinal symptoms), if increased purulence of sputum was one of the two symptoms. 

The treatment of exacerbations with antibiotics was commenced empirically, following the official GOLD recommendations. In cases with a positive result from microbiological evaluation of sputum, the treatment was continued following the identification of bacterial sensitivity to certain antibiotics. Oral corticosteroids were given as needed (a dose of 40 mg oral prednisone per day for five days). The course of exacerbation was evaluated as a function of resolution of symptoms, and the treatment was considered to be successful if cure or clinical improvement was achieved. 'Cure' was defined as complete resolution of the cardinal symptoms, whereas 'clinical improvement' was defined as a return of symptoms to their baseline severity [11,12,24].

### 2.7. Data Collection (Daily Diary Card)

Similar to the study on BE and exacerbation indices carried out by Patel et al. [7], all study subjects maintained daily diary cards where they noted any appearance of increase in the intensity of major symptoms (dyspnea, sputum amount, and sputum purulence) or minor symptoms (nasal discharge/congestion, sore throat, wheezing, cough, etc.) over their chronic (stable) symptoms. A member of the study team met with study subjects within 48 hours of the detection of deterioration in symptoms, and diagnosis was confirmed for each case. Exacerbation and its resolution were defined as mentioned above. Exacerbation number, their duration, and the duration of exacerbation-free intervals (i.e., a period of time between two exacerbations) were calculated for each of the study subjects, based on data from diary cards for a 12-month period of follow-up.

### 2.8. Statistical Analysis

Statistical analysis was performed using the Statistical Package for the Social Sciences (SPSS) version 11.0 for Windows (SPSS Inc., Chicago, IL, USA). Continuous variables were expressed as mean values with standard deviation (SD), and the nominal variables as numbers and percentages. Analyses of the data included testing the differences in prevalence and comparison of the means by chi-square testing (or Fisher’s exact test where appropriate), and the *t*-test for independent-samples. A *p*-value less than 0.05 was considered to be statistically significant.

## 3. Results

In 64 outpatients referred from primary care to the Institute for Occupational Health of R. Macedonia, Skopje, in the period September–November 2015, the diagnosis of COPD was established and they were classified as Group D according to the combined disease assessment. Fifty-four of these newly diagnosed COPD patients were enrolled in the study. Four patients refused to participate in the study, three did not complete the daily diary card adequately, in two patients an exacerbation occurred at the start of the study period, and in one patient there was no consensus in the interpretation of HRCT.

Demographic characteristics of the study subjects are shown on Table 1.

Over the study period, 151 exacerbations were documented, 84 (55.6%) from COPD patients with BE, and 67 (44.4%) from COPD patients without BE. A total of 123 out of 151 exacerbations (81.4%) met criteria of bacterial exacerbations being treated with oral antibiotics, 67 (54.5%) from COPD patients with BE (79.7% of all exacerbations in these patients) and 56 (45.5%) from COPD patients without BE (83.5% of all exacerbations in these patients). In addition, 36 bacterial exacerbations (23.8%) from COPD patients with BE were treated with oral prednisolone, 22 (26.1%) from COPD patients with BE, and 14 (20.9%) from COPD patients without BE. Exacerbation from 14 COPD patients (9.3%) required hospital treatment, eight (9.5%) from the COPD patients with BE, and six (8.9%) from COPD patients without BE.

**Figure 1:**
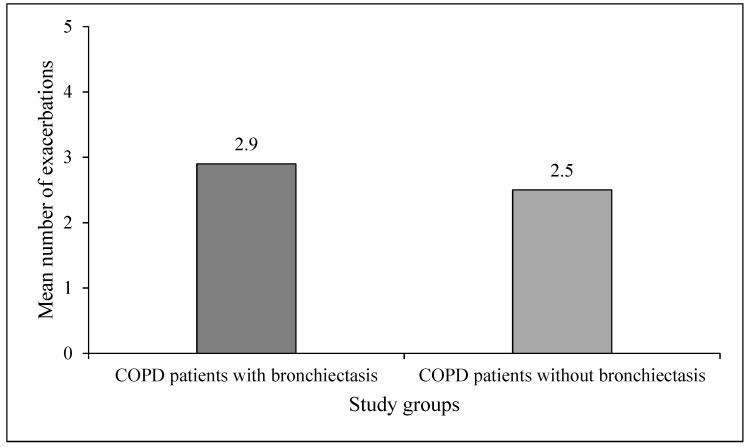
Mean number of exacerbations in the study groups.

A mean number of exacerbations over the study period were significantly higher in COPD patients with BE (2.9 ± 0.5; ranging from two to four) as compared to their number in COPD patients without BE (2.5 ± 0.3; ranging from two to three) (*p* = 0.0008) (Figure 1).

**Figure 2:**
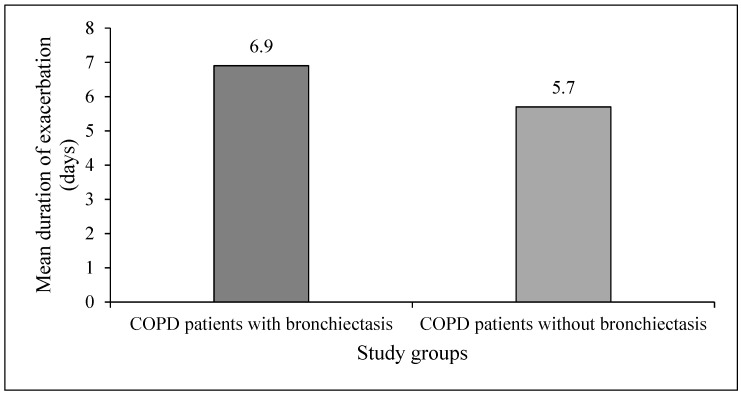
Mean duration of exacerbations in the study groups.

The mean duration of exacerbations (expressed in days needed for cure or clinical improvement, i.e., complete resolution of symptoms or return of symptoms to their baseline severity) in the COPD patients with BE (6.9 ± 1.8 days; ranging from 5–10 days) was significantly longer than the mean duration of exacerbations in the COPD patients without BE (5.7 ± 1.4 days; ranging from 4–8 days, *p* = 0.0085) (Figure 2).

**Figure 3:**
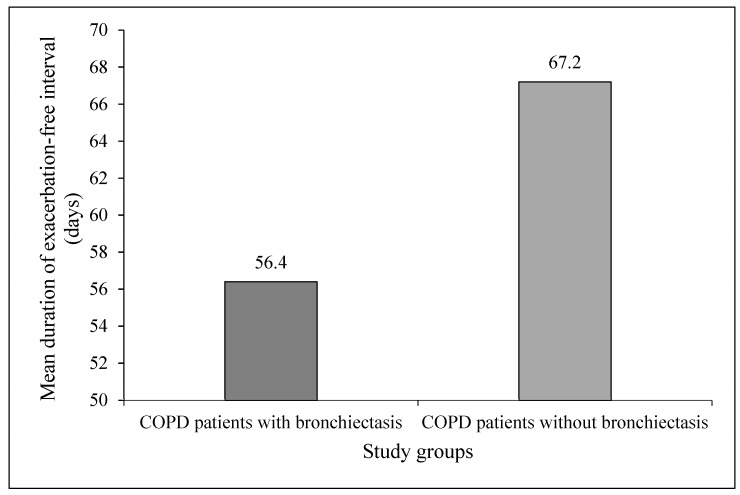
Mean duration of exacerbation-free interval in the study groups.

The mean duration of exacerbation-free interval (expressed in days) in the COPD patients with BE (56.4 ± 17.1; ranging from 37–82 days) was significantly shorter than the COPD patients without BE over the same time period (67.2 ± 14.3; ranging from 49–88 days, *p* = 0.0149) (Figure 3).

## 4. Discussion

As mentioned earlier, there is a clear association between BE and COPD, termed the BE-COPD overlap syndrome (BCOS) [5,25]. In addition, the results of several studies indicate that BE is more frequent in COPD patients with more severe airflow limitation [26]. However, some studies with a large number of COPD patients, such as the Evaluation of COPD Longitudinally to Identify Predictive Surrogate Endpoints (ECLIPSE) cohort (more than 2000 study subjects), reported a frequency of BE of 5% among study subjects with severe COPD and a frequency of 7% among study subjects with very severe COPD, although the fact that patients with other pulmonary conditions were excluded may have affected the results [27]. Patients with COPD are prone to exacerbations, which account for significant morbidity and mortality, as well as significant worsening of quality of life. Lower airway bacterial colonization is a common clinical finding in COPD, and is increasingly being recognized as an independent stimulus for airway inflammation. Patients with COPD and coexisting BE have greater bronchial inflammation and greater chronic colonization of bronchial mucosa by a potentially pathogenic microorganism, this can lead to more frequent exacerbations with longer duration [28,29]. In addition, there is evidence for significant increase of the incidence of hospital admissions for patients with BE as a secondary diagnosis (the most frequent primary diagnosis was COPD) in a 10-year period (2004–2013), as opposed to cases of BE as the primary diagnosis [30].

In the present study, we compared the frequency and duration of exacerbations and exacerbation-free intervals between a Group D COPD patients with BE as confirmed by HRCT, and Group D COPD patients who were confirmed negative for BE. Both study groups had similar demographic characteristics. Similar to results from previous studies on COPD patients in both study groups, we found a high prevalence of active smokers, a low proportion of ex-smokers, and a high proportion of subjects exposed to ETS [31,32].

As mentioned earlier, exacerbations are important events in the course of COPD because they have a negative impact on all aspects of the disease. The most common cause of COPD exacerbations is believed to be bacterial respiratory infection. In addition, some patients with COPD are particularly prone to exacerbations, and they are defined as 'frequent exacerbators' [33]. One of the reasons for more frequent exacerbations in COPD patients may be due to the coexisting BE in these patients. We registered a higher frequency of exacerbation, as well as a higher frequency of exacerbations which met criteria for bacterial exacerbation, in the COPD patients with BE than in the COPD patients without BE. Our findings indicated a significantly higher mean number of exacerbations, and a significantly higher mean duration, as well as a significantly shorter exacerbation-free interval in COPD patients with BE than in COPD patients without BE. Results from a meta-analysis of fourteen observational studies comparing COPD characteristics in patients with or without coexisting BE, indicated a two-fold higher risk of exacerbations in COPD patients with comorbid BE, than in COPD patients without BE [34]. Conversely, results by Martinez-Garcia et al. indicated that factors independently associated with the presence of BE in patients with moderate to severe COPD were severe airflow obstruction, isolation of potentially pathogenic microorganisms from sputum, and at least one hospital admission for exacerbation in the previous year, i.e., that the number of exacerbations and bacterial colonization in the airways were not related to BE [35].

The reasons why the presence of BE in COPD can be related to more frequent and longer durations of exacerbations are speculative. The presence of bacteria in the lower airways in COPD patients impairs host defense mechanisms, which results in epithelial cell integrity disruption and inflammation, impaired mucociliary clearance, further airway structural damage, which could be the mechanism for more frequent, and more severe COPD exacerbations [36]. In addition, incomplete resolution of bacterial infection or bacterial colonization is considered as a risk for relapse; appropriate antibiotic treatment of bacterial infection in COPD exacerbation should be important for the prevention of relapses and the delay of subsequent exacerbations [37,38].

The present study must be interpreted within the context of its limitations. First, the relatively small number of the subjects in the study groups could have certain implications on the data obtained and its interpretation. Second, the study groups included only Group D COPD patients, which could also have certain implications on data obtained and its interpretation. Third, a 12-month period is a relatively short follow-up period, and this may also have impacted the study results. The strength of the study is in its detailed approach to identifying the characteristics of exacerbations (frequency and duration of exacerbation and duration of exacerbation-free interval) in COPD patients with and without BE.

## 5. Conclusions

In conclusion, in a study aimed at comparing the frequency and duration of bacterial exacerbation, as well as the duration of the exacerbation-free interval, between Group D COPD patients with BE and Group D COPD patients who were confirmed negative for BE, we found non-significantly higher mean numbers, significantly higher mean durations, and significantly shorter mean exacerbation-free intervals in COPD patients with BE. Our findings support the theory that early identification of patients with COPD and BE would be an important advance, as it will provide opportunities to start an appropriate treatment.

## Figures and Tables

**Table 1:** Demographics of the study subjects.

Variable	COPD patients with BE (*n* = 27)	COPD Patients without BE (*n* = 27)	*p*-Value *
M/F ratio	1.2	1.4	
M (%)	15 (55.5%)	16 (59.2%)	0.783
Mean age (years)	53.3 ± 4.1	52.5 ± 4.8	0.513
Mean BMI (kg/m^2^)	26.1 ± 2.8	25.7 ± 2.1	0.555
Mean duration of COPD (years)	9.2 ± 3.1	8.9 ± 3.4	0.736
Mean values of spirometric parameters (% predicted)			
FVC	68.3 ± 7.8	70.6 ± 5.4	0.213
FEV_1_	44.1 ± 4.2	46.3 ± 3.9	0.061
FEV_1_/FVC ratio	0.64 ± 0.02	0.65 ± 0.01	0.093
Microbiological evaluation of sputum in stable patients			
Negative result	11 (40.7%)	18 (66.7%)	0.056
*Haemophylus influenzae*	9 (33.3%)	6(22.2%)	0.362
*Moraxella catarrhalis*	2 (7.4%)	3 (11.1%)	0.500
*Streptococcus pneumoniae*	1 (3.7%)	/	/
*Pseudomonas aeruginosa*	4 (14.8%)	/	/
Treatment of stable COPD			
LA β_2_-agonist + ICS	24 (88.9%)	25 (92.6%)	0.639
LA anticholinergic	20 (74.1%)	19 (70.4%)	0.761
Oral theophyline	4 (14.8%)	5 (18.5%)	0.715
Bronchiectasis type		/	/
Cylindrical	15 (55.5%)	/	/
Varicose	12 (45.5%)	/	/
Cystic	/	/	/
Smoking status			
Active smokers	9 (33.3%)	8 (29.6%)	0.769
Ex-smokers	15 (55.5%)	14 (51.8%)	0.785
Never smokers	3 (11.1%)	5 (18.5%)	0.352
Exposed to ETS	12 (44.4%)	10 (37.1%)	0.579
Comorbidities			
Arterial hypertension	8 (29.6%)	7 (25.9%)	0.761
Musculoskeletal disorders	5 (18.5%)	6 (22.2%)	0.735
Ischemic heart disease	5 (18.5%)	4 (14.8%)	0.715
Diabetes mellitus type 2	3 (11.1%)	3 (11.1%)	1.000

Numerical data are expressed as mean value with standard deviation; frequencies as number and percentage of study subjects with certain variable. * Tested by Chi-square test (or Fisher’s exact test where appropriate) and Independent-samples *t*-test. BE: bronchiectasis; COPD: chronic obstructive pulmonary disease; M: male; F: female; BMI: body mass index; kg: kilogram; m: meter; % pred.: % of the predicted value; FVC: forced vital capacity; FEV_1_: forced expiratory volume in one second; LA: long-acting; ICS: inhaled corticosteroid; ETS: environmental tobacco smoke.
